# Nutrition Support for Children with Paediatric Intestinal Pseudo-Obstruction (PIPO) in the United Kingdom—An Explorative Survey by the British Society of Paediatric Gastroenterology, Hepatology and Nutrition (BSPGHAN)

**DOI:** 10.3390/nu18101575

**Published:** 2026-05-15

**Authors:** Alessandra Mari, Keith James Lindley, Sally Buxton, Sian Kirkham, Jutta Kӧglmeier

**Affiliations:** 1 Department of Paediatric Gastroenterology, Great Ormond Street Hospital for Children NHS Foundation Trust, London WC1N 1EH, UK; 2 Paediatric Department, V. Buzzi Children’s Hospital, 20154 Milan, Italy; 3 Department of Paediatric Gastroenterology, Great North Children’s Hospital, Newcastle upon Tyne NE1 4LP, UK; 4 Department of Paediatric Gastroenterology, Queen’s University Hospital, Nottingham NG7 2UH, UK

**Keywords:** paediatric intestinal pseudo-obstruction, artificial enteral nutrition, parenteral nutrition, oral intake, texture specific diet, bite-and-dissolve foods, outcome

## Abstract

**Background/Objectives**: Paediatric intestinal pseudo-obstruction (PIPO) is a disorder of gut motility in childhood, frequently leading to intestinal failure (IF). Consensus on optimum nutrition management (oral, enteral, intravenous feeding exclusively or in combination) is lacking. Our aim was to investigate the current approach to the nutrition support of children (<18 years) with PIPO managed in Gastroenterology centres in the United Kingdom (UK) and long-term mode of feeding. **Methods**: An electronic questionnaire was sent to the members of the British Society of paediatric Gastroenterology, Hepatology and Nutrition (BSPGHAN). The data collected (October–November 2023) included patient demographics, disease phenotype, age at symptom onset and mode/type of feeding. **Results**: A total of 36 patients fulfilled criteria for PIPO; 22/36 (61.1%) patients were female, and 25/36 (69.4%) were white British. A total of 15/36 (41.6%) became symptomatic during the neonatal period and 23/36 (63.8%) within the first year of life. A total of 5/36 (13.9%) was eating a normal solid diet: 2/36 (5.5%) of these never required artificial feeding, 2/36 (5.5%) were started on a normal diet after short-term parenteral nutrition (PN) in the first year of life, and 1/36 (2.8%) re-established oral eating after 10 years of total PN following small bowel transplantation. A total of 31/36 (86.1%) required permanent artificial feeding (enteral and/or parenteral) after an average time of symptoms of 14 months. A total of 2/36 (5.5%) was exclusively on enteral nutrition (EN), and 4/36 (11.1%) was on total PN. A total of 25/36 (69.4%) received a combination of PN and oral diet (normal, or bite and dissolve, or normal but minimal intake) and/or EN. **Conclusions**: The results show that how and with what children with PIPO are fed in the UK varies widely. Larger studies are needed to make evidence-based recommendations on the best nutrition approach.

## 1. Introduction

Paediatric intestinal pseudo-obstruction (PIPO) is a rare gastrointestinal motility disorder with an estimated incidence of 1:40,000 to 1:100,000 [1,2,3]. Affected children present with symptoms suggestive of intestinal obstruction, but a mechanical blockage cannot be established. Unfortunately, many children still undergo multiple laparotomies before the diagnosis is made. In the UK, a national service for the diagnosis and management of paediatric patients with suspected PIPO was hence commissioned in 2012 to reduce the number of unnecessary surgeries in these children. Referral of children to the nationally commissioned hospital based in London by all other UK paediatric Gastroenterology centres is expected by National Health Service (NHS) Commissioning. Despite earlier diagnosis, the progression to intestinal failure (IF) and long-term PN dependency is still frequently seen. PIPO is different from chronic intestinal pseudo-obstruction (CIPO) in adults, who usually develop CIPO due to an underlying systemic disorder. In contrast to this, most children have congenital forms [4]. A PIPO diagnosis can be made, if two of the four 2018 European Society of Paediatric Gastroenterology, Hepatology and Nutrition (ESPGHAN) criteria summarised in Table 1 are present [4].

Despite better diagnostic tools, treatment remains largely supportive. Dietary modification, the use of enteral and parenteral nutrition, prokinetics, antibiotics for small intestinal bacterial overgrowth and, where appropriate, surgery are the cornerstones of management [3]. Many children and their families report poor quality of life with frequent admissions to hospital [5,6].

Poor enteral tolerance renders a significant number of patients with PIPO dependent on parenteral nutrition (PN). Whilst this allows for improvement in nutrition status and normal growth and development, PN is no panacea and remains associated with a number of complications [7]. Compared to other causes of intestinal failure, mortality is much higher in these children, with as many as four to 30% of patients not surviving into adulthood [4,8,9]. Reasons for this are that PIPO is a progressive and diffuse intestinal neuromuscular disorder rather than a structural problem seen in congenital enteropathies or short bowel syndrome. Children with PIPO have a higher risk of central venous catheter-associated sepsis, intestinal failure-associated liver disease and renal problems due to severe fluid and electrolyte shifts associated with the pooling of fluid in distended, non-motile segments of bowel. These patients should hence be managed by multidisciplinary teams with expertise in paediatric intestinal failure rehabilitation.

However, a consensus on the best nutrition approach in these young patients is lacking. Whilst some promote early, often exclusive PN, others allow oral solid intake as long as possible even if intravenous feeding has become necessary. Some centres advise parents to offer bite-and-dissolve foods instead of normal textures. Artificial enteral nutrition with liquid feeds given through a gastric or jejunal tube is also used alongside oral and/or parenteral nutrition [3].

The aim of this cross-sectional study was to investigate current nutrition strategies used by IF rehabilitation units in children and adolescents with PIPO in the UK and, if the results allowed, to understand if one was superior compared to others, to inform the best possible practice. The results could aid in developing a consensus guideline with implications not only for the management of British children with PIPO but also to those outside the UK.

## 2. Materials and Methods

An electronic questionnaire was designed (Microsoft word 365) by the lead investigating centre of this study and sent to UK centres caring for children with PIPO via BSPGHAN. It included 25 questions, either multiple choice or short free-text style, asking about disease phenotype (according to the 2018 ESPGHAN criteria) age at onset, investigation results, feeding regime (oral normal, oral texture-modified, artificial enteral, parenteral nutrition in isolation or combined), re-introduction of oral/enteral feeding after a period of total/partial PN and general demographic data. The full questionnaire is available in the Supplementary Materials. Patients who did not fulfil the ESPGHAN diagnostic criteria currently considered the gold standard for diagnosis PIPO were excluded. Parenteral nutrition was considered to be partial when providing less than 75% of energy requirements. Oral intake was labelled ‘minimum’ when small amounts of food were taken, either in liquid or solid form, but their nutritional value/amount of calories was too small to contribute to the child’s nutritional intake. Bite-and-dissolve diet was defined as the intake of a texture-modified diet of foods that dissolves in the mouth to a liquid or very loose puree on contact with saliva. An example would be potato snacks made from potato starch and deep-fried, known in the UK for their ‘melt-in-the-mouth’ texture.

Patient data were collected between October 2023 and December 2023. The questionnaires were filled in by health professionals of the individual UK paediatric units who had children with PIPO on their case load and sent to the lead investigating unit. Information collected was then entered anonymously into the database. Collected data were summarised with descriptive statistics. Ethical review and approval were waived for this study, as no additional intervention was performed on the human subjects involved. The study was registered with the Institutional Research and Development department of the lead investigating centre as clinical audit.

## 3. Results

Forty-two questionnaires were submitted by paediatric Gastroenterologists working at three large paediatric Gastroenterology centres in the UK caring for children and adolescents with PIPO. Two questionnaires were not included into the data analysis as the patients did not meet ESPGHAN criteria for PIPO. A further four were excluded as they referred to children seen in two centres and were hence duplicates (a questionnaire was completed for these children by a paediatric Gastroenterologist in each of the two centres and hence two questionnaires were submitted for four children). Thirty-six questionnaires were therefore included in the study. Of these, twenty-nine were completed by paediatric Gastroenterologists working at the National Commissioned Group (NCG) referral centre for children with PIPO based in London and included patients from 13 centres, with six from a large paediatric Gastroenterology unit working in collaboration with the NCG hospital in the North of England and one from a centre in the East Midlands. Each questionnaire contained anonymous data of a single child with PIPO.

**Figure 1:**
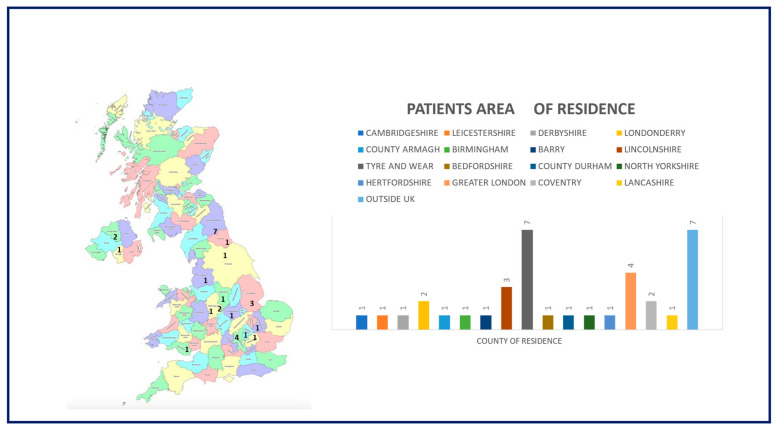
Patient demographics—area of patient residence.

Twenty-two children (61.1%) were females. The most common ethnic background was Caucasian (83.3%), followed by Arabic (8.3%) and Asian (2.7%). For two children, no information on ethnicity was provided. As some patients were sent to the nationally commissioned centre for PIPO from other European countries, not all children were resident in the UK: 2/36 lived in the Netherlands (5.5%), 2/36 in the Republic of Ireland (5.5%), 1/36 in Austria (2.7%), 1/36 in Germany (2.7%) and 1/36 in Bulgaria (2,7%). The remaining twenty-nine children (80.5%) lived in the UK (see Figure 1).

More than half of the children (63.8%) developed symptoms within the first year of life, and, among these, 15/36 (4.6%) developed symptoms within the first month after birth; 6/36 children (16.6%) became symptomatic between 1 and 3 years of age, 4/36 (11.1%) between 4 and 6 years of age, and 3/36 children (8.3%) after 7 years of age. The majority of patients (34/36; 94.4%) were unable to maintain adequate growth and nutritional status on an oral diet at the time of diagnosis. The median time from the onset of symptoms and when the patient was no longer tolerating diet by mouth was four months. Antro-duodenal manometry (ADM) was performed in almost all patients (33/36; 91.6%). In 29/36 children, neuropathic changes were found (80.5%), 4/36 (11.1%) had a neuromyopathic pattern. A total of 22/36 children (61.1%) had persistent or recurrently dilated loops of small intestine. Only 5/36 (13.8%) of the patients had genetic mutations and/or metabolic abnormalities associated with PIPO. Mutations in the ACTG2 gene were the most common genetics found in 3/36 (8.3%).

Other investigations including nuclear medicine transit studies and histopathology were available in 12/36 (33.3%) and 5/36 (13.8%) of patients, respectively. These are summarised in Table 2.

A total of 2/36 patients (5.5%) continued to eat food by mouth and did not need additional artificial enteral or parenteral nutrition support during the course of their illness. This was due to their ability to maintain adequate growth and/or nutritional status on an oral diet alone. Temporary artificial feeding became necessary in 3/36 children (8.3%). Out of these, 1/36 (2.7%) was fully re-established on an exclusive oral diet after intestinal transplantation, when gut function was rehabilitated. In 1/36 (2.7%), oral food was gradually reintroduced over time and PN weaned during the first year of life with regular support by a team experienced in the management of the disease. Therefore, at the time of completion of the questionnaire, 5/36 of patients (13.8%) managed an exclusively oral diet.

**Figure 2:**
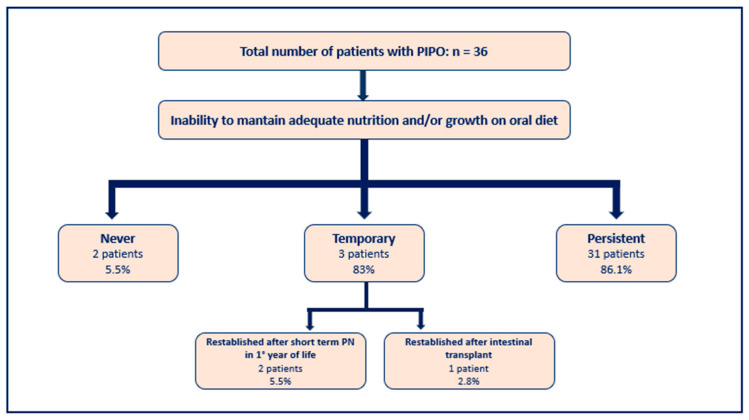
Inability to maintain adequate nutrition and/or growth on oral diet.

Long-term artificial enteral and/or parenteral nutrition was required in 31/36 (86.1%) of children (see Figure 2).

**Figure 3:**
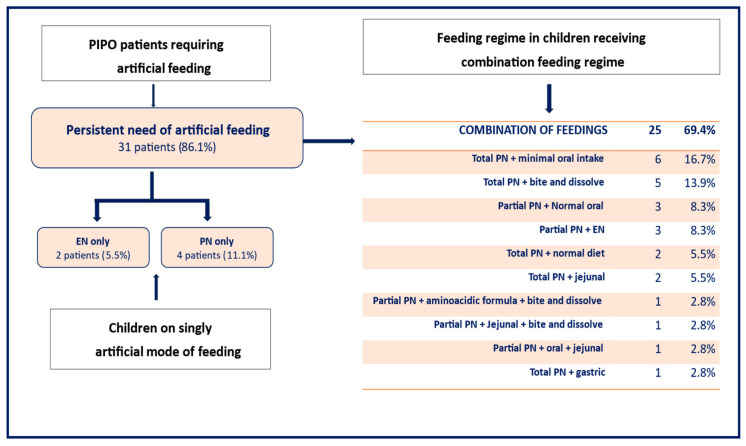
Mode of artificial feeding of UK children with PIPO.

The type of feeding used in these patients varied significantly. Four patients (4/36; 11.1%) were managed with total PN and did hence not receive any oral food or enteral feeds via a feeding device. Two children (2/36; 2.7%) were managed with exclusive artificial enteral nutrition, one with a gastric feeding tube and one with jejunal feeds. The remaining twenty-five (25/36; 69.4% of the total) received a combination feeding regime as shown in Figure 3.

In our cohort, a total of 29/36 (80.5%) of the children received PN, with either partial PN (9/36, 25%) or total PN (20/36, 55.5%). The average time between the onset of symptoms and the start of PN (partial or total) was 2 years. Only 4/36 patients (11.1%) managed to re-establish some oral intake, which was gradually increased over time; in 2/36 (0.72%), PN could hence be discontinued, while the other two (0.72%) were able to increase their oral intake but continued to depend on partial PN.

## 4. Discussion

Since the publication of the 2018 diagnostic criteria for differentiating PIPO clearly from adult-onset CIPO, the knowledge of this rare disease has increased and allowed for a diagnosis to be made in much younger children [1,4,10,11]. However, only little data is available in the literature on how to manage children with PIPO in the best possible way in order to rehabilitate those with poor nutrition and to guarantee normal growth and development.

The focus of the European Reference Network for rare inherited and congenital anomalies (ERNICA) is dedicated to gastrointestinal disorders in paediatric patients in need of specialised long-term and surgical care [12]. It connects 52 expert healthcare providers in 21 European Union member states. The group published data on the diagnosis and management of children with PIPO in 2023 [13].

Unfortunately, only little information regarding the best nutrition practice of these young patients was included. Our study results describe for the first time the different approaches used to manage the nutrition of children with PIPO in different units across the UK. Our data can therefore inform a future dialogue about optimal nutrition strategies for children with PIPO.

Eating is an essential part of human life, not only to guarantee survival; it also hassignificant psychosocial implications. Not allowing a child to eat a normal oral diet over the fear of potential complications, including food-related bezoar formation, dysmotility-associated nausea, and vomiting and bloating aggravated by eating, may lead to potentially over-cautious limitations of the oral intake of young PIPO patients. Not being able to engage in normal family meals or eating with friends at nursery or school can negatively impact affected children and their families [14]. Understanding what the best feeding strategy was according to age and presentation of the child as well as the type of PIPO could not only improve outcomes but also reduce disease burden.

Patient numbers and age at presentation in our cohort reflect those of previous publications. More than half of the children (63.8%) and hence the majority developed symptoms within the first year of life, as published in the past [1,3,7].

Unlike age at presentation, the proportion of children with PIPO who required partial or total PN in hospital or required long-term home PN has differed amongst previous authors.

In data published more than 20 years ago, between 60% and 75% of patients, respectively, were prescribed either partial or total PN and only a small proportion received some form of oral and/or enteral nutrition throughout the course of their disease [8,9]. In a more recent cohort of 54 children with PIPO, more than half (57%) were successfully weaned of intravenous nutrition support with the re-establishment of intestinal autonomy and only 41% remained on home PN during a median follow up period of 17.5 years [14]. The authors found that the progression to irreversible intestinal failure leading to long-term PN dependence occurred more frequent in children whose PIPO symptoms began in the neonatal period [15].

On the other hand, in a group of 62 Japanese paediatric patients with PIPO, 29% ate a normal diet by mouth, and only 10% were managed with total parenteral nutrition [2].

In a cohort of 58 Chinese children, none ever received long-term PN. However, due to differences in healthcare provision available in China, home PN could not be offered to all patients included. The authors themselves were concerned that some patients were therefore weaned off PN prior to discharge from hospital even though they did not tolerate full enteral and/or oral nutrition and therefore became malnourished when PN was discontinued [11].

In our cohort of patients managed in UK centres, most children with PIPO (29/36; 80.5%) received PN as part of their medical management—either as partial PN (9/36, 25%) or total PN (20/36, 55.5%)—during the study period. Of these, just over half (19/36: 52.8%) still tolerated some form of oral intake. In six out of these 19 patients, oral intake was minimal and hence not considered contributing to their overall calorie intake but allowed for comfort. This shows that even in those children who were dependent on full intravenous calories and often also fluid support, a small amount of oral intake is worth considering, as this may contribute to the child’s and the family’s quality of life.

Only two (5.5%) children were managed with exclusive artificial enteral nutrition via a feeding device, whilst a quarter of children (9/36) children required PN in addition to artificial enteral nutrition. Although parenteral support was clearly needed in the majority of our paediatric PIPO patients, a large number continued on some form of oral intake (either normal solid food or texture adjusted) and/or liquid enteral feeds in addition to PN. Remarkably, in 3/36 (8.3%) of patients who had stopped tolerating any form of oral intake, oral tolerance gradually improved again over time and with careful management within the setting of a multidisciplinary IF clinic.

Trying to use the gut and allowing children to continue or to restart eating can help to reduce both the risk of long-term PN complications, as well as the burden of disease. Although the number of children whose oral tolerance improved over time was small and it was hence not possible to understand why this was possible in some children with PIPO and not others, this appeared achievable in some patients with PIPO.

Interestingly, when comparing children with confirmed gene mutations associated with PIPO (4/36; 11%), the ability to tolerate enteral/oral nutrition varied widely. One child completely lost enteral tolerance soon after birth, one as a young child and two beyond the 10th birthday. Although the number is too small to draw conclusions, genetic factors may not necessarily influence/be exclusively responsible for enteral tolerance despite children with genetic mutations associated with PIPO often starting to show symptoms at a young age. Feeding practices used amongst centres caring for paediatric PIPO patients in the UK differed widely, except for the use of parenteral nutrition in the majority of patients, with an increasing need as children became older. The reasons behind may be disease progression with worsening intestinal function, but also oral aversion to food as a consequence of having been on PN for years, previously bad experience with an attempt to restart/continue feeds, as well as the centre’s individual approach to these patients.

The amount and type of diet consumed by children (25/36; 69.4%) who were eating by mouth in addition to receiving PN were very variable in our cohort: 11/36 (30.5%) ate various amounts of normal solids and 7/36 (19.4%) were only offered bite-and-dissolved foods. Some clinicians believe firmly in the avoidance of solids over concerns about the risk of phytobezoar formation associated with very impaired gastrointestinal motility. A solid bezoar could complicate PIPO further by the event of a true mechanical obstruction [16].

It is hence often practice to manage these patients with a carbohydrate and fat-restricted diet consisting of small meals low in soluble and insoluble fibres. In some cases, the texture of the diet is modified and limited to only bite-and-dissolve food [3,17]. However, the true evidence behind the rationale for this practice is limited and often purely based on expert opinion. It is hence difficult to understand if this texture specific diet was only advised over fear of causing complications, or the child did simply not tolerate normal consistency food.

It is also important to mention that 2/36 (5.5%) children in our study never lost the ability to maintain adequate nutrition and/or growth on an oral diet alone. In 3/36 patients, an oral diet and discontinuation of parental support were successful. Only one patient (2.8%) who re-established enteral autonomy (2.8%) underwent multivisceral intestinal transplantation as a form of intestinal rehabilitation. The majority of affected children (31/36: 86.1%) started and remained on some form of artificial nutrition at some point during the course of their disease. It would be helpful to understand if these patients had permanent loss of total or partial gut function, or if attempts to reintroduce enteral and/or oral feeds were never made again once the PIPO diagnosis had been made and artificial nutrition support was started.

A periodical reassessment, at least every 12 months, to see if a young PIPO patient is able tolerate at least some enteral and/or oral feeds appears a reasonable approach to us. Particularly after a period of gut rest, e.g., following stoma formation, previous reports in the literature have shown that some children can tolerate more enteral intake after ileostomy surgery. Decompression of the bowel may hence not only contribute to improvement in symptoms but also improved gut function [18].

Our study has limitations. As PIPO is a rare disorder, our cohort was, although of similar size to previous publications, relatively small and consisted only of thirty-six children. Results were based on information collected from questionnaires filled in by the patients’ responsible clinicians based at different centres and not all data may have hence been provided to the lead investigator. In addition, it is possible that not all children and adolescents with PIPO living in the UK were identified by the survey, as their respective responsible clinicians may have chosen not to submit a questionnaire. The study centre did not receive any questionnaires or referrals from centres in Scotland during the study period. However, since the start of the NCG service for children in 2012, referral of children to the nationally commissioned hospital based in London by all other UK paediatric Gastroenterology centres is expected by National Health Service (NHS) Commissioning. The majority of children reported by the NCG service were already referred by other UK cenres and hence known and included by the paediatric Gastroenterologists at the NCG hospital, including children from across England, Wales and Northern Ireland. Although all patients included had a diagnosis made based on the ESPGHAN definition for PIPO, only two out of four criteria are required to be eligible. Although the majority of children lost the ability to maintain adequate growth and nutrition with an exclusively oral diet, other diagnostic criteria differed. This was at least in part dependent on access to specialist investigations such as manometry catheter investigations in older children being diagnosed before the start of the nationally commissioned diagnostic services for PIPO in 2012.

A full diagnostic work-up (antro-duodenal manometry, nuclear medicine study and histopathology) was not done for every patient in the cohort. However, it is still possible that children may have had variable phenotypic presentations. It was hence not possible to determine if the nutrition approach varied between different PIPO phenotypes. As only few patients had reported disease-associated genetic mutations, we could also not draw conclusions if oral/enteral tolerance was associated with certain PIPO genes as it is unknown if genetic testing was negative or simply not carried out in the majority of the patients included.

However, the main objective of this study was to investigate what nutritional management strategies are used by paediatric Gastroenterology teams caring for children and adolescents with PIPO in the UK. We aimed to get better insight into the best possible nutritional approach to these patients. Our results demonstrate that there is no uniform approach and consensus on how these children should be fed is lacking.

## 5. Conclusions

How children with PIPO are fed in the UK varies significantly between centres. Whilst some young patients continue a normal oral diet, others are prescribed different combinations of enteral and parenteral nutrition or exclusive PN. Enteral tolerance in children with PIPO can improve in some, allowing for a reduction in PN. Discontinuation of intravenous nutrition support may be possible in a small number of patients if managed by a multidisciplinary intestinal failure rehabilitation team with expertise in nutrition management of these patients. Which group of patients falls into this category could not be identified in our survey. We hence suggest re-assessing the tolerance of enteral nutrition in children managed with parenteral nutrition periodically in close collaboration with a paediatric dietitian at least every 12 months. In our view, continuing oral food, even in small quantities, should be allowed if clinically tolerated, as this will contribute to improved quality of life in these children.

Larger prospective multi-centre studies are needed to get a better understanding of the best nutritional approach for each PIPO phenotype as well as the impact of pharmacological agents such as prokinetics and venting stomas on enteral and oral feeding tolerance. This would inform the development of an evidence-based consensus guideline for the management of these patients in and outside the UK.

## Figures and Tables

**Table 1:** Diagnostic criteria for PIPO according to ESPGHAN.

Diagnosis of PIPO if at least two of the following apply for two months after birth or six thereafter:	
I	Objective measure of small intestinal neuromuscular involvement (histopathology, transit study, manometry)
II	Recurrent and/or persistently dilated loops of small intestine with air fluid levels
III	Genetic and/or metabolic abnormality definitely associated with PIPO
IV	Inability to maintain adequate nutrition and/or growth on oral feeding (thus requiring enteral and/or parenteral nutrition support)

**Table 2:** Diagnostic criteria based on ESPGHAN definition used for PIPO diagnosis.

Criteria	n (%)	Findings
**Inability to maintain adequate growth and/or nutritional status on oral feeding**	**34/36 (94.4%)**	-
Persistent/recurrent dilated loops of small intestine	22/36 (61.1%)	-
Genetic/metabolic abnormality associated with PIPO	5/36 (11.1%)	ACTG2 gene mutation 3/36 (8.3%)
		Other 2/36 (5.5%)
**Objective measure of small bowel neuromuscular involvement**	**Antro-duodenal manometry 33/36 (91.6%)**	**Neuropathic alterations 29/36 (80.5%)**
		Neuromyopathic alterations 4/36 (11.1%)
	Nuclear medicine studies 12/36 (33.3%)	Delayed gastric emptying 8/36 (22.2%)
		Delayed small bowel transit 4/36 (11.1%)
	Histopathology 5/36 (13.8%)	Myopathic alterations 3/36 (8.3%)
		Neuropathic alterations 1/36 (2.7%)
		Neuromyopathic alterations 1/36 (2.7%)

## Data Availability

The data presented in this study are available on request from the corresponding author due to privacy.

## Supplementary Materials

The following supporting information can be downloaded at https://www.mdpi.com/article/10.3390/nu18101575/s1. Supplement S1. Questionnaire.

## Abbreviations

The following abbreviations are used in this manuscript:

BSPGHAN	British Society of Paediatric Gastroenterology, Hepatology and Nutrition
PIPO	Paediatric intestinal pseudo-obstruction
IF	Intestinal failure
UK	United Kingdom
PN	Parenteral nutrition
CIPO	Chronic intestinal pseudo-obstruction
EN	Enteral nutrition
ESPGHAN	European Society of Paediatric Gastroenterology, Hepatology and Nutrition

## References

[1] Batra S., Rahman S., Rana M.S., Matta S., Darbari A. (2020). Epidemiology and healthcare utilization of inpatient admissions in children with pediatric intestinal pseudo-obstruction. Neurogastroenterol. Motil..

[2] Muto M., Matsufuji H., Tomomasa T., Nakajima A., Kawahara H., Ida S., Ushijima K., Kubota A., Mushiake S., Taguchi T. (2014). Pediatric chronic intestinal pseudo-obstruction is a rare, serious, and intractable disease: A report of a nationwide survey in Japan. J. Pediatr. Surg..

[3] Pescarin M., Day H., Thapar N., Jackman L., Saliakellis E., Lindley K.J., Nikaki K., Hill S., Kӧglmeier J., Rybak A. (2023). Optimizing nutrition IN pediatric intestinal pseudo-obstruction syndrome. Neurogastroenterol. Motil..

[4] Thapar N., Saliakellis E., Benninga M.A., Borrelli O., Curry J., Faure C., De Giorgio R., Gupte G., Knowles C.H., Staiano A. (2018). Paediatric Intestinal Pseudo-obstruction: Evidence and Consensus-based Recommendations from an ESPGHAN-Led Expert Group. J. Pediatr. Gastroenterol. Nutr..

[5] Viti F., De Giorgio R., Ceccherini I., Ahluwalia A., Alves M.M., Baldo C., Baldussi G., Bonora E., Borrelli O., Dall’oGlio L. (2023). Multi-disciplinary Insights from the First European Forum on Visceral Myopathy 2022 Meeting. Dig. Dis. Sci..

[6] Jackman L., Arpe L., Thapar N., Rybak A., Borrelli O. (2024). Nutritional Management of Pediatric Gastrointestinal Motility Disorders. Nutrients.

[7] Hartman C., Shamir R., Simchowitz V., Lohner S., Cai W., Decsi T., ESPGHAN/ESPEN/ESPR/CSPEN Working Group on Pediatric Parenteral Nutrition (2018). ESPGHAN/ESPEN/ESPR/CSPEN guidelines on pediatric parenteral nutrition: Complications. Clin. Nutr..

[8] Mousa H., Hyman P.E., Cocjin J., Flores A.F., Di Lorenzo C. (2002). Long-term outcome of congenital intestinal pseudoobstruction. Dig. Dis. Sci..

[9] Faure C., Goulet O., Ategbo S., Breton A., Tounian P., Ginies J.-L., Roquelaure B., Despres C., Scaillon M., Maurage C. (1999). Chronic intestinal pseudoobstruction syndrome: Clinical analysis, outcome, and prognosis in 105 children. Dig. Dis. Sci..

[10] Connor F.L., Di Lorenzo C. (2006). Chronic intestinal pseudoobstruction: Assessment and management. Gastroenterology.

[11] Tang P., Lu L., Yan W., Tao Y., Feng H., Cai W., Wang Y. (2023). Long-term follow-up for pediatric intestinal pseudoobstruction patients in China. Nutr. Clin. Pract..

[12] https://www.ern-ernica.eu.

[13] Mutanen A., Demirok A., Wessel L., Tabbers M. (2023). Pediatric intestinal pseudo-obstruction: An international survey on diagnostic and management strategies in the European Reference Network for Rare Inherited and Congenital Anomalies intestinal failure teams. J. Pediatr. Gastroenterol. Nutr..

[14] Turcotte M.-C., Faure C. (2022). Pediatric intestinal pseudoobstruction: Progress and challenges. Front. Pediatr..

[15] Diamanti A., Fusaro F., Caldaro T., Capriati T., Candusso M., Nobili V., Borrelli O. (2019). Pediatric intestinal pseudo-obstruction: Impact of neonatal and later onset on clinical and nutritional outcomes. J. Pediatr. Gastroenterol. Nutr..

[16] Perisic V.N., Scepanovic D., Radlovic N. (1995). Intestinal pseudoobstruction and phytobezoar. J. Pediatr. Gastroenterol. Nutr..

[17] Mari A., Lindley K.J., Guz-Mark A., Hilberath J., Hojsak I., Norsa L., Tabbers M., Thomassen R.A., Verduci E., Kӧglmeier J. (2025). Nutrition practices of children with paediatric intestinal pseudo-obstruction in Europe—A survey by the network for intestinal failure rehabilitation and transplantation in Europe. J. Pediatr. Gastroenterol. Nutr..

[18] Pakarinen M.P., Kurvinen A., Koivusalo A.I., Ruuska T., Mäkisalo H., Jalanko H., Rintala R.J. (2013). Surgical treatment and outcomes of severe pediatric intestinal motility disorders requiring parenteral nutrition. J. Pediatr. Surg..

