# Supplementary material for: Nutrition Support for Children with Paediatric Intestinal Pseudo-Obstruction (PIPO) in the United Kingdom—An Explorative Survey by the British Society of Paediatric Gastroenterology, Hepatology and Nutrition (BSPGHAN)
Source: Nutrients. 2026 May 15;18(10):1575. doi: 10.3390/nu18101575 (PMC13209299; doi:10.3390/nu18101575)
Supplement: Supplementary file 1 [file nutrients-18-01575-s001.zip › nutrients-4237371-supplementary.pdf]

NUTRITION PRACTICES OF CHILDREN WITH PAEDIATRIC INTESTINAL PSEUDO-OBSTRUCTION IN THE UNITED KINGDOM – A SURVEY BY THE BRITISH SOCIETY OF PAEDIATRIC GASTROENTEROLOGY, HEPATOLOGY AND NUTRITION (BSPGHAN)

Questionnaire

a) Demography

1. Sex: male □ female □
2. Month and year of birth:
3. Ethnic background:

   White British □ white other □

   Asian □ Black, Caribbean or African □

   Mixed Arabic □ any other □
4. County of residence (eg Hertfordshire/England)

b) Disease phenotype

1. Age of onset of first symptoms:
2. Age of onset when ability to maintain adequate nutrition by mouth was no longer possible:
3. Objective measure of small intestinal neuromuscular involvement

   a) Manometry

   b) Histopathology

   c) motility MRI

d) nuclear medicine transit study

4. Recurrent and/or persistently dilated loops of small intestine with air fluid levels

   Yes ☐ no ☐

5. Genetic and/or metabolic abnormalities definitively associated with PIPO (eg ACTG2 mutation)

   Yes ☐ → mutation:

   Metabolic abnormalities:

   No ☐

Nutrition

1. Oral feeding

   Yes ☐

   a) Normal diet ☐
   b) Exclusion diet (eg dairy free): ☐ → free from:
   c) Special consistency (eg bite and dissolve or purees) ☐ → type:

   No ☐

2. Liquid enteral nutrition (gastric or postpyloric enteral feeding)

   Yes ☐ → nasogastric tube ☐ gastrostomy ☐

   nasojejunal tube ☐ gastrojejunostomy ☐ jejunostomy ☐

   no ☐

3. Parenteral nutrition (PN)

   yes ☐ → partial ☐ total ☐ nights per week: age at start of PN:

   no ☐

4. Combination of any of the above

Yes □ → combination of:

No

5. Has enteral/oral intake been re-established/increase at any point after a period of Parenteral Nutrition

   Yes □ → if so why (eg after ileostomy formation):

   No □

6. Nutrition status:

a) Weight: Kg
b) height/length: cm

   if available:
c) Mid upper arm circumference: cm

THANK YOU VERY MUCH FOR COMPLETING THIS QUESTIONNAIRE

Please return questionnaires to:
Jutta Koeglmeier
Consultant Paediatric Gastroenterologist
Great Ormond Street Hospital for Children NHS Foundation Trust London

Email: Jutta.Koeglmeier@gosh.nhs.uk
